# *Plumeria alba*-Mediated Green Synthesis of Silver Nanoparticles Exhibits Antimicrobial Effect and Anti-Oncogenic Activity against Glioblastoma U118 MG Cancer Cell Line

**DOI:** 10.3390/nano12030493

**Published:** 2022-01-30

**Authors:** Muthuraj Rudrappa, Hassan Ahmed Rudayni, Rasha Assad Assiri, Asmatanzeem Bepari, Dhanyakumara Shivapoojar Basavarajappa, Shashiraj Kariyellappa Nagaraja, Bidhayak Chakraborty, Pallavi Sathyanarayana Swamy, Shekappa Ningappa Agadi, Shaik Kalimulla Niazi, Sreenivasa Nayaka

**Affiliations:** 1 Department of Studies in Botany, Karnatak University, Dharwad 580003, Karnataka, India; rmuthuraj20@gmail.com (M.R.); dhanyakumarsb@gmail.com (D.S.B.); rajscbz@gmail.com (S.K.N.); pallabchakraborty3@gmail.com (B.C.); pallavi.gupnar@gmail.com (P.S.S.); shekaragadi@rocketmail.com (S.N.A.); 2 Department of Biology, College of Science, Imam Muhammad bin Saud Islamic University, Riyadh 11623, Saudi Arabia; harudayni@imamu.edu.sa; 3 Department of Basic Medical Sciences, College of Medicine, Princess Nourah Bint Abdulrahman University, Riyadh 11671, Saudi Arabia; RAAssiri@pnu.edu.sa (R.A.A.); ambepari@pnu.edu.sa (A.B.); 4 Department of Preparatory Health Sciences, Riyadh Elm University, Riyadh 12611, Saudi Arabia

**Keywords:** *Apocynaceae*, *Plumeria alba*, nanotechnology, nano-antibiotics, chemotherapeutics, cytotoxic, apoptosis

## Abstract

*Plumeria alba* (*P. alba*) is a small laticiferous tree with promising medicinal properties. Green synthesis of nanoparticles is eco-friendly, cost-effective, and non-hazardous compared to chemical and physical synthesis methods. Current research aiming to synthesize silver nanoparticles (AgNPs) from the leaf extract of *P. alba* (P- AgNPs) has described its physiochemical and pharmacological properties in recognition of its therapeutic potential as an anticancer and antimicrobial agent. These biogenic synthesized P-AgNPs were physiochemically characterized by ultraviolet-visible spectroscopy, Fourier-transform infrared spectroscopy (FTIR), scanning electron microscopy (SEM), transmission electron microscope (TEM), atomic force microscopy (AFM), X-ray diffractometry (XRD), and zeta potential analysis. Antimicrobial activity was investigated against *Escherichia coli*, *Pseudomonas aeruginosa*, *Enterobacter aerogenes*, *Enterococcus faecalis*, *Bacillus subtilis*, *Streptococcus pneumoniae*, *Candida albicans*, and *Candida glabrata*. Anticancer activity against glioblastoma U118 MG cancer lines was investigated using an MTT assay, and apoptosis activity was determined by flow cytometry. UV–visible spectroscopic analysis portrayed surface plasmon resonance at 403 nm of synthesized P-AgNPs, and FTIR suggested the presence of amines, alkanes, and phenol molecules that could be involved in reduction and capping processes during AgNPs formation. Synthesized particles were spherical in shape and poly-dispersed with an average particle size of 26.43 nm and a poly-dispersity index (PDI) of 0.25 with a zeta potential value of −24.6 mV, ensuring their stability. The lattice plane values confirm the crystalline nature as identified by XRD. These P-AgNPs exhibited potential antimicrobial activity against selected human pathogenic microbes. Additionally, the in vitro MTT assay results showed its effective anticancer activity against the glioma U118 MG cancer cell line with an IC_50_ value of 9.77 µg/mL AgNPs by initiating apoptosis as identified by a staining study with flow cytometric Annexin V–Fluorescein Isothiocyanate (FITC) and Propidium Iodide (PI). Thus, *P. alba* AgNPs can be recommended for further pharmacological and other biological research. To conclude, the current investigation developed an eco-friendly AgNPs synthesis using *P. alba* leaf extract with potential cytotoxic and antibacterial capacity, which can therefore be recommended as a new strategy to treat different human diseases.

## 1. Introduction

Nanotechnology is the science that converges on the synthesis, characterization, exploration, and application of nanosized (1–100 nm) materials in diverse fields of applied science ranging from material science to biotechnology [1]. The physicochemical properties of nanomaterials are quite different from those of bulk materials because of their extremely small size and high surface volume ratio. Thus, they exhibit new, improved properties based on specific characteristics such as size, distribution, and morphology [2]. Currently, it is one of the most demanding research disciplines that has attracted substantial interest from chemists, biologists, physicists, and engineers for various applications in emerging technologies and consumer products such as biomedicine, renewable energy, agriculture, antibacterial purposes, optical, sensors, catalytic devices, electronic appliances, and other products used for personal care, water, and soil treatment [3]. It enables the production of various nanoparticles (NPs) from various sources, including metals, metal oxides, non-metals, lipids, polymers, and numerous nanocomposites. NPs are classified into two main groups: organic and inorganic NPs. The organic NPs include micelles, dendrimers, liposomes, and hybrid and compact polymeric NPs. The inorganic NP group includes fullerenes, quantum dots, silica, and noble metal as NPs [4]. Inorganic nanoparticles, especially those that are purely created from gold, silver, and copper, have unique optical and photothermal properties due to their well-known localized surface plasmon resonance (LSPR) [5]. Thus, NPs from noble metals have achieved much significance over the last few years because of their applicability in biology, material science, medicine, physics, and chemistry. NPs can be synthesized using three methods, namely physical, chemical, and biological approaches. The chemical and physical approaches for the synthesis of nanoparticles are technically difficult and expensive. Reducing agents such as sodium borohydride, N,N-dimethylformamide, trisodium citrate, and other chemicals used in the chemical methods of synthesizing metal nanoparticles can increase the cost of production and result in hazardous wastes being released into the environment [6].

Green synthesis of nanoparticles using environmentally friendly substances is an emerging branch in nanotechnology. Recently, inspired by the perception of green chemistry, the biological synthesis of nanoparticles has been focused on utilizing biological entities, such as plants, algae, and micro-organisms. Green synthesis offers numerous advantages, such as eco-friendliness, low production cost, energy efficiency, and compatibility for biomedical and pharmaceutical applications as compared with chemical synthesis [7]. Many plants have been used and recorded for the efficient and extracellular synthesis of copper, silver, and gold nanoparticles such as *Azadirachta indica*, *Nelumbo nucifera*, *Tinospora cordifolia*, *Mimosa pudica geranium*, *Musa paradisiacal*, *Putranjiva roxburghii* Wall. *Cinnamon zeylanicum*, *Cinnamon camphora*, *Tamarind* leaf extract, *Aloe vera* plant extracts, *Phyllostachys* sp., leaves extract, and *Acalypha indica* [8,9,10]. Out of all the noble metal nanoparticles, silver nanoparticles (AgNPs) have gained a unique focal point because of their typical properties, such as chemical stability, good electrical conductivity, and catalytic and antibacterial activity [11]. AgNPs have diverse applicability in fields of drug discovery, luminescence, renewable energy technologies, textiles, the food industries, electronics, optics, cosmetics, catalysis, wound dressing, dentistry, and agriculture [12]. Green synthesized AgNPs have been reported to have various biological properties including anti-inflammatory, antidiabetic, antiplasmodial, anticancer, and antimicrobial characteristics. The use of NPs in many products such as soap, food, and textiles, and coating on surgical devices increases their market value [13]. Among biological methods for synthesizing nanoparticles of silver, microbe-mediated synthesis of nanoparticles is not industrially reasonable as it requires maintenance of highly aseptic conditions and proper maintenance of microbes. Therefore, using plant extracts to synthesize nanoparticles over microorganisms is potentially advantageous because of the straightforward, scaled-up procedure; it produces less biohazardous materials, and the elaborate process of maintaining cell culture is not required [11]. Moreover, plants contain several phytochemicals with complicated chemical structures; such medicinal plants have shown great ability in forming AgNPs with desired therapeutic actions [14]. AgNPs are also popular anticancer agents; they can initiate programmed cell death by disrupting membrane integrity, cellular functions, causing damage to the nucleus membrane, genetic mutations, and toxicity [15]. There are scarce reports on the biofabrication of AgNPs using *Plumeria alba (P. alba)* and its biological activity [6]. Therefore, we selected *P. alba.*

*Plumeria* or frangipani is a member of a genus of flowering plants in the *Apocynaceae* family. The plants are innate to Mexico, Central America, and South America and reach as far South as Brazil and the Caribbean; they can be grown in tropical and sub-tropical regions [16]. *Plumeria alba* is a laticiferous small tree or shrub. It is grown as an ornamental plant in gardens because of its fragrant flower. The leaves are lanceolate to oblanceolate; the flowers are white in color and fragrant in corymbose fascicles. Its parts such as bark, leaves, latex, and flowers engage in various biological activities such as antibacterial, antioxidant, antitumor, antiarthritic, and antidiabetic activities [17]. It bears edible fruit; the seeds have demonstrated hemostatic properties. Latex has been used to treat herpes, ulcers, and scabies, and the powdered bark has been used as a plaster applied on hard tumors [18], while the other species of the *Plumeria* are used for cardiotonic, purgative, hypotensive, and diuretic applications [19]. The plant *P. alba* contains many bioactive compounds such as β-sitosterol, mixtures of amyrins, amyrinacetate, iridoids, isoplumericin, scopotetin, plumieride coumerate, plumieride, and plumieride coumerate glucoside [20,21].

Gliomas are the most common primary central nervous system (CNS) tumors. Among these, half of all new diagnoses are represented by glioblastoma (GBM), the most malignant type of brain cancer with a poor prognosis and a high mortality rate, usually within 15–18 months of diagnosis [22]. Current strategies for treating GBM involve chemotherapy, radiation therapy, and surgical removal of the tumor [23], which are not well tolerated and have not significantly increased the survival rate in diagnosed glioma patients [24]. The main drawback of glioma treatments is the ability of these cancer cells to infiltrate and migrate rapidly [25]. Thus, finding the crucial sources of novel lead structures for discovering anticancer agents from natural products and nanoscience technology due to their diverse ‘drug-like structure’ and ‘biologically friendly’ molecular qualities is urgently needed. Nowadays, nanoparticles with chemotherapeutics have gained interest in cancer treatment [26]. Another global concern is the increased appearance of multi-resistant bacterial pathogens that have become a worldwide problem [27]. Therefore, to study potential clinical applications of *P. alba,* we aimed to explore its ability to synthesize AgNPs.

Objectives of the study:

The aim of the study was to synthesize nanoparticles from *P. alba* leaf extract, characterize them by using different analytical methods, and test their antimicrobial and anticancer biological activities. Therefore, the following aspects of the *P. alba* leaf extract were investigated:

(a) Green synthesis of AgNPs using *P. alba* aqueous leaf extract (P-AgNPs);
(b) Characterization of the synthesized AgNPs by using various analytical techniques such as UV–visible spectroscopy, FTIR, XRD, SEM, EDX, zeta potential, and particle size;
(c) Determination of the antimicrobial activity of P-AgNPs against human pathogenic microorganisms;
(d) Evaluation of the anticancer activity of P-AgNPs by employing the in vitro cytotoxic assay method (MTT assay) against the U118 MG glioma cancer cell line;
(e) Exploration of the mechanism of action of P-AgNPs against the U118 MG glioma cancer cell line using flow cytometric analysis.

## 2. Materials and Methods

### 2.1. Preparation of Extract

**Figure 1:**
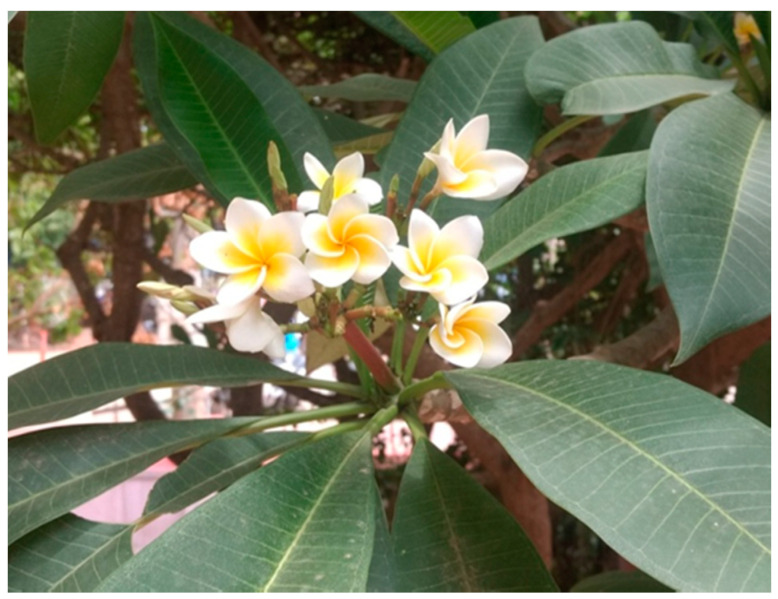
*Plumeria alba* plant habitat.

Fresh leaves of *P. alba* (Figure 1) were collected in Jayanagar, Dharwad, Karnataka, India. The plant identity was confirmed by experts from the Department of Botany, Karnatak University, Dharwad. Then, the leaves surface was cleaned by running tap water to remove debris and other contaminated organic contents, followed by aqueous extraction using double-distilled water. A total of 20 g of the *P. alba* leaves was finely cut into small pieces and added to the conical flask containing 100 mL of distilled water. Then, it was kept in a water bath for 1 h at 60 °C. Later, the extract was cooled down and filtered through Whatman No. 1 filter paper. Finally, the obtained plant extract was stored in a refrigerator at −4 °C for further use.

### 2.2. Biosynthesis of AgNPs from the Extracts of Plumeria alba

The *P. alba* leaf extract was mixed with 1 mM AgNO_3_ solution in the ratio of 1:5 and it was kept for incubation for 24 h in the dark. The synthesis of P-AgNPs was confirmed by a color change from pale yellow to reddish-brown due to reduction of Ag^+^ to Ag^0^. Then, the mixture was centrifuged at 5000 rpm for 20 min; the pellet was collected and dried in a hot air oven, and synthesized P-AgNPs were kept for further analysis [28].

### 2.3. Characterization of Synthesized P-AgNPs

#### 2.3.1. UV–Visible Spectroscopy Analysis of Synthesized P-AgNPs

UV 9600A UV/Visible Spectrophotometer (Shanghai Metash Instruments Co., Ltd., Shanghai, China) was used to analyze the biologically synthesized P-AgNPs from leaf extract of *P. alba* for the absorption maxima at 300 to 600 nm. The graph was plotted as wavelength (X-axis) against absorbance (Y-axis).

#### 2.3.2. Analysis of Surface Functional Groups and Compounds: FTIR Analysis of Synthesized P-AgNPs

The transmittance FTIR analysis of synthesized nanoparticles was carried out to determine the probable functional groups involved in the capping and proficient stabilization of nanoparticles. The P-AgNPs were dried in a thermostatted desiccator at 45 °C for 24 h to avoid the presence of water molecules and thoroughly ground with potassium bromide (about 5% of P-AgNPs) and pressed to make thin discs. The spectrum of AgNPs from *P. alba* leaf extract was recorded in the transmittance mode between 400 cm^−1^ and 4000 cm^−1^ at a resolution of 4 cm^−1^ using NICOLET 6700 FTIR Spectrophotometer (Waltham, MA, USA).

#### 2.3.3. Scanning Electron Microscopic Analysis of Synthesized P-AgNPs

Shape, morphology, and nature of distribution of synthesized AgNPs were analyzed by scanning electron microscope (SEM), and silver presence was confirmed by energy dispersive spectroscopy (EDX). The sample for SEM analysis was prepared by placing a very small quantity of AgNPs on sticky conductive carbon tape mounted on aluminum stub followed by gold sputtering for 3 min. The imaging and elemental analysis was carried out through scanning electron microscope (JSM-IT 500LA, Tokyo, Japan).

#### 2.3.4. Transmission Electron Microscopic Analysis of Synthesized P-AgNPs

Size and surface morphology of synthesized P-AgNPs were determined by transmission electron microscope. A drop of AgNPs solution from *P. alba* leaf extract was applied to the surface of carbon copper grid. The images were recorded at 6000 X to 8000 X magnification by the instrument Hitachi, (Model: S-3400N, CA, USA) at the voltage of 80 kV. The poly-dispersity of the nanoparticles population was expressed in PDI by calculating the average radius and standard deviation of 18 nanoparticles. PDI was calculated as

p = σ/R_Avg_ (1)

where p = dispersity, σ = standard deviation of a radius of batch of nanoparticles, and R_Avg_ = average radius of nanoparticles.

#### 2.3.5. Atomic Force Microscopic Analysis of Synthesized P-AgNPs

Morphology, size, and distribution of P-AgNPs were characterized through atomic force microscopy analysis. The AgNPs suspension in distilled water was prepared by ultrasonication for 5 min. A very thin film of nanoparticles suspension was applied on clean glass slides and allowed to dry in room temperature for 10 h. The slides were then scanned with an oscillated cantilever attached with the instrument Nanosurf Flex AFM. (Liestal, Switzerland.)

#### 2.3.6. X-ray Diffractometric Analysis of P-AgNPs

The crystalline structure, lattice parameters, and crystalline grain size of synthesized AgNPs were analyzed using Rigaku Miniflex 600 (Austin, TX, USA) for the X-ray diffractometry. The powdered sample of AgNPs was kept in cavity slide and gently pressed to make a smooth surface. The diffractometer instrument was operated with the help of data scan software at a scan rate of 1.2° per min. The XRD spectra were recorded between 5° and 80° by the X-ray diffractometer equipped with CuKα filter (λ = 0.15418 nm), 2ϴ/ϴ scanning mode. The obtained diffractogram was compared to the standard JCPDS card No. 04-0783. The size of the NPs was determined by Scherrer’s formula given as follows:

D = 0.89λ/βcosθ (2)

where λ is the X-ray wavelength, D is the particle size (nm), β is the full line width at half maximum (FWHM) elevation of the important peak, and θ is the refractive (Bragg’s) angle.

#### 2.3.7. Zeta Potential Analysis and Size Distribution of Synthesized P-AgNPs

Zeta potential analysis was carried out to estimate the surface charge of synthesized P-AgNPs, which was used as a reference for the physical stability of nanoparticles. The sample suspension was prepared in distilled water with 2 to 3 rounds of subsequent ultrasonication and later centrifuged at 5000 rpm for 15 min. The viscosity of the dispersion medium was 0.875 mPa.s. The electric voltage was set at 3.4 V with a conductivity of 0.130 mS/cm. The obtained supernatant was again diluted 4–5 times with distilled water, and then the particle size distribution was characterized by nano-analyzer instrument (Horiba Scientific Nanoparticle Analyzer SZ-100, Kyoto, Japan).

### 2.4. Biological Activity of Synthesized P-AgNPs

#### 2.4.1. Antimicrobial Activity of Synthesized P-AgNPs

The antimicrobial activity of synthesized P-AgNPs was measured against selected pathogenic organisms by the agar well diffusion method in triplicates. The test organisms included three Gram-negative bacteria: *Escherichia coli* (MTCC40), *Pseudomonas aeruginosa* (MTCC9027), and *Enterobacter aerogenes* (MTCC2822); three Gram-positive bacteria: *Enterococcus faecalis* (MTCC6845), *Bacillus subtilis* (MTCC6633), and *Streptococcus pneumoniae* (MTCC1935); and two fungal strains: *Candida albicans* (MTCC227) and *Candida glabrata* (MTCC3019), which were procured from MTCC, Pune. A total of 1mg/ml of P-AgNPs suspension was prepared using sterilized distilled water. Selected pathogenic organisms were cultured overnight in Muller Hinton broth (Mumbai, India). Antimicrobial activity was carried out on nutrient agar plates; pre-cultured pathogenic organisms in 0.5 McFarland concentrations were swabbed on separate nutrient agar plates, and 6 mm wells were produced using sterilized cork borer. The wells were filled with 100 µL of positive control (streptomycin for bacteria and amphotericin-B for fungi, 25 µg/mL each), 100 µL of negative control (sterilized distilled water) and 25, 50, and 100 µL of P-AgNPs suspension for each organism. All the plates were incubated for 24 h at 37 °C, and after the incubation period zone of inhibition was recorded [29].

#### 2.4.2. Anticancer Activity of Synthesized P-AgNPs from *P. alba* Leaf Extract

The anticancer activity of the synthesized P-AgNPs was evaluated by MTT assay against U118 MG cancer cell lines. The U118 MG human brain cancer cell lines were procured from NCCS Pune, India. DMEM (Dulbecco’s Modified Eagle Medium) high-glucose medium (#AL111, HiMedia, Mumbai, India) was used to culture the cell lines supplemented with 10% fetal bovine serum (FBS, #RM10432, Himedia, Mumbai, India), penicillin (100 IU/mL), streptomycin (100 μg/mL), and amphotericin-B (5 μg/mL) in a humidified incubator of 5% CO_2_ at 37 °C till confluence occurred. In the exponential growth period, these cell lines were washed, dissociated with trypsin (0.2%), and resuspended in complete culture media. A total of 200 µL of cell suspension was seeded in 96-well plates at the required cell density of 20,000 cells per well, followed by incubation at 37 °C and 5% CO_2_ for 24 h. Then, the P-AgNPs suspension with different concentrations of 6.25 µg/mL, 12.5 µg/mL, 25 µg/mL, 50 µg/mL, and 100 µg/mL was added to the culture medium, and cisplatin with a concentration of 6 µg/mL was taken as positive control depending upon its anticancer properties in GBM [26,30]. The plate was incubated for 24 h at 37 °C with an atmosphere of about 5% CO_2_, followed by the addition of 50 μL of MTT reagent to make up the final volume of 0.5 mg/mL. The plates were covered with aluminum foil to avoid exposure to the light and incubated for 4 h. A total of 100 μL dimethyl sulfoxide (DMSO) (#PHR1309, Sigma Aldrich, Bangalore, India) solubilization solution was added to each well to dissolve the formazan format on the last step. The absorbance was recorded at 570 nm by the ELISA reader, and the IC_50_ value was calculated by using a linear regression equation, i.e., Y = Mx + C, where Y = 50, M, and C values were derived from the viability graph [31,32]. Three independent experiments were performed.

#### 2.4.3. Apoptosis Assay of P-AgNPs by Flow Cytometry

The synthesized P-AgNPs were subjected to examine the rate of cell death in U118 MG cancer cell line. Cultured U118 MG cancer cells were taken in 96-well plate with a density of 0.5 × 10^6^ cells/2 mL. The cells were incubated in a CO_2_ incubator overnight at 37 °C for 24 h. After the incubation period, the cells were treated with IC_50_ concentrations of biosynthesized P-AgNPs suspension for 24 h. The control experiment was performed in 2 mL of culture medium without any treatment of P-AgNPs suspension. At the end of the treatment, the treated cells were washed twice with phosphate-buffered saline (PBS). Later, PBS was discarded, and 200 μL of the trypsin–ethylenediaminetetraacetic acid (EDTA) solution was added and incubated at 37 °C for 3–4 min. Then, 2 mL of culture medium was added. The cells were harvested directly into 12 × 75 mm polystyrene tubes and centrifuged for five min at 300 rpm at 25 °C. The supernatant was removed carefully, followed by washing the cells with PBS twice and removing PBS completely. A total of 5 μL of Fluorescein Isothiocyanate (FITC)–Annexin V was added to the obtained pellet with a gentle vortex and incubated for 15 min at 25 °C in the dark, followed by the addition of 5 μL of Propidium Iodide (PI) and 400 μL of 1X binding buffer and analysis by flow cytometry [33].

### 2.5. Statistical Analysis

P-AgNPs images were chosen for characterization from one of the triplicates. The results of antimicrobial and anticancer experiment are expressed as the means ± standard deviation (SD, for each group n = 3) to produce graph preparation. Statistical significance at *p* < 0.05 between the different antimicrobial groups was evaluated by one-way ANOVA analysis of variance.

## 3. Results

### 3.1. Green Synthesis of P-AgNPs and Their Characterization

**Figure 2:**
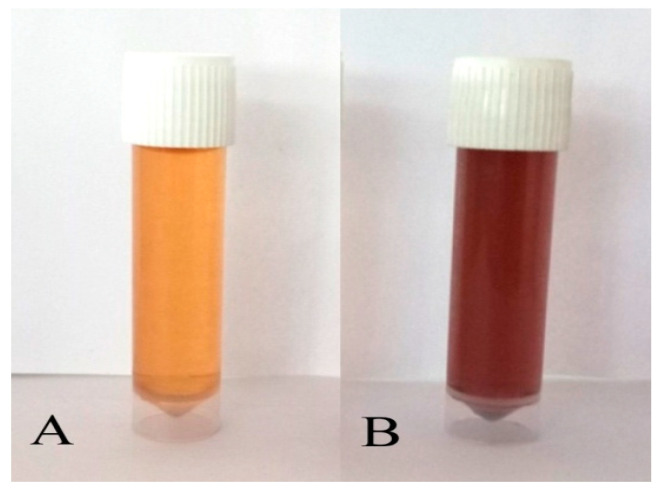
Biosynthesis of P-AgNPs from leaf extract of *P. alba*: Mixture of aqueous *Plumeria alba* leaf extract and AgNO_3_ solution (**A**) before incubation and (**B**) after incubation.

**Figure 3:**
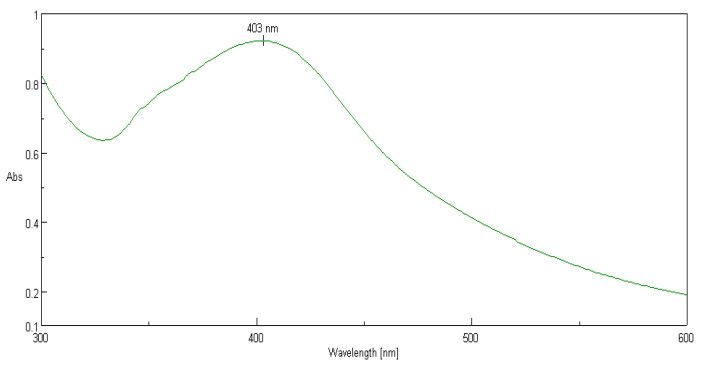
UV–visible analysis of synthesized P-AgNPs from *P. alba* leaf extract.

The present study was aimed at synthesizing AgNPs from a *P. alba* leaf aqueous extract; the synthesis was confirmed by a change in the color of extract from pale yellow to reddish-brown suspension after mixing with 1 mM AgNO_3_ (1:5 *v*/*v*) and incubation for 24 h (Figure 2). Furthermore, the UV–visible spectroscopic analysis confirmed the production of AgNPs after the incubation period of 24 h in the dark chamber with an absorption peak observed at a wavelength of 403 nm (Figure 3), which is the characteristic feature of surface plasmon resonance (SPR), as it confirmed the synthesis of AgNPs.

**Figure 4:**
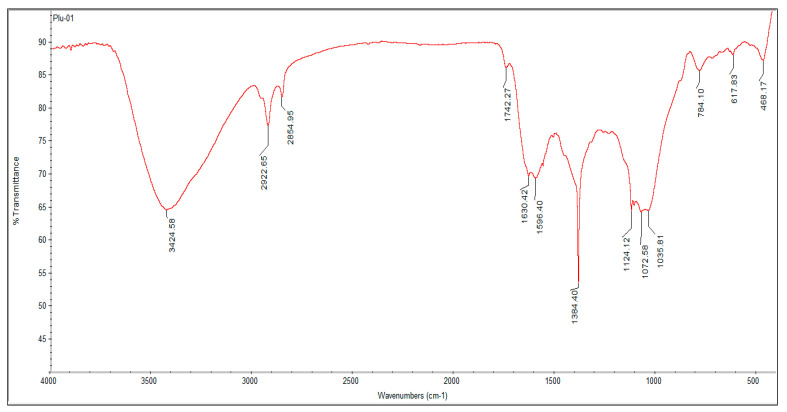
FTIR analysis of synthesized P-AgNPs from *P. alba* leaf extract.

The FTIR analysis of synthesized AgNPs was carried out to analyze the functional groups involved in the synthesis of AgNPs. The FTIR analysis of green synthesized AgNPs revealed many absorption peaks between 400 cm^−1^ and 4000 cm^−1^. Peaks were obtained at 3424, 2922, 2854, 1742, 1630, 1596, 1384, 1124, 1072, 1035, 784, 617, and 468 cm^−1^ (Figure 4). The strong and broad peak at 3424 cm^−1^ displayed the presence of O-H stretching of the alcohol group, and the peak values at 2922 and 2854 cm^−1^ indicated the C-H bond stretching of the alkane group. The band observed at 1742 cm^−1^ affirmed the stretching esters with a C=O bond, and the intense peaks at 1630, 1596, and 1384 cm^−1^ confirmed the C=C stretching of alkene, N-H bending of amine, and O-H bending of phenols, respectively. The presence of secondary and primary alcohol groups with C-O stretching was confirmed by the peaks at 1124 and 1072 cm^−1^. The peak at 1035 cm^−1^ was associated with the S=O stretching of the sulfoxide group, and the peaks at 784, 617, and 468 cm^−1^ were associated with C-I and C-Br stretching of halo compounds.

**Figure 5:**
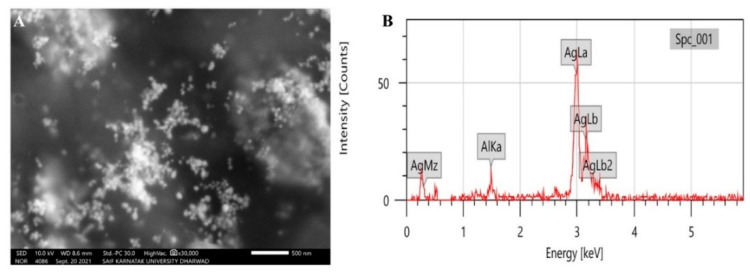
SEM with EDX analysis of synthesized P-AgNPs from *P. alba* leaf extract (**A**) SEM image showing surface morphology, (**B**) EDX spectrum indicating the presence of silver.

**Figure 6:**
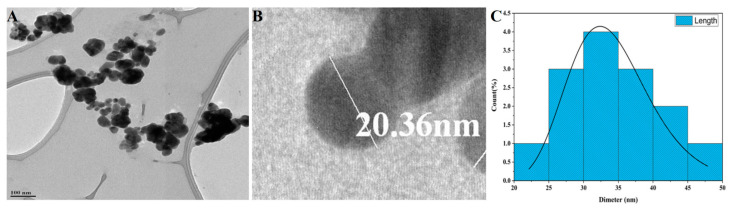
TEM Analysis of Synthesized P-AgNPs from *P. alba* leaf extract. (**A**) Polydispersed AgNPs, (**B**) Single spherical-shaped AgNP and (**C**) P-AgNPs’ histogram showing size distribution.

**Figure 7:**
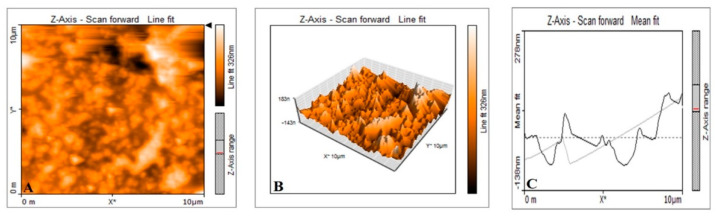
AFM analysis of synthesized P-AgNPs from *P. alba* leaf extract. (**A**) Two dimensional depiction, (**B**) size distribution, and (**C**) histogram.

The morphological analysis of synthesized P-AgNPs was carried out by SEM analysis. The SEM image is displayed in Figure 5A, which showed the spherical-shaped and poly-dispersed nature of P-AgNPs. The elemental analysis of P-AgNPs by EDX expressed a strong absorption peak of silver at the energy level of 3 keV and confirmed silver as a major constituent present in the nanoparticles because it performed the major role as the stabilizing and reducing agent in synthesis (Figure 5B). TEM images of *P. alba* nanoparticles showed the poly-dispersed nature of particles. However, few of the synthesized P-AgNPs were agglomerated, and the size of these particles ranged from 20.12 to 45.40 nm with a PDI value of 0.25 (Figure 6A). Figure 6B depicts a spherical-shaped nanoparticle with a diameter of 20.36 nm, and the size distribution is shown in Figure 6C. The shape, size, and surface morphology of the synthesized nanoparticles were also analyzed by AFM. Figure 7A shows that the particles were spherical in shape with a particles size of 18.23 to 53.68 nm. Figure 7B,C indicate the size distribution and histogram of synthesized P-AgNPs, respectively.

**Figure 8:**
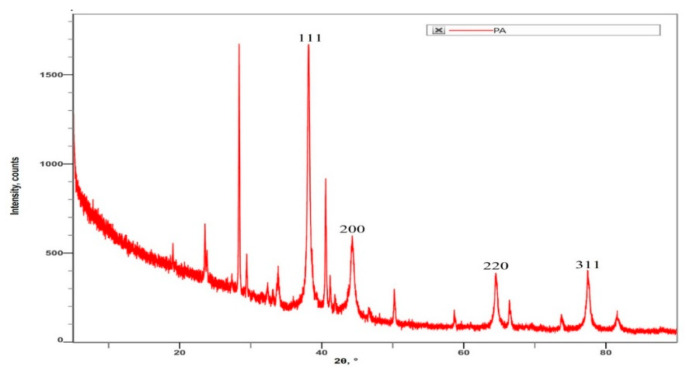
XRD analysis of synthesized P-AgNPs from leaf extract of *P. alba*.

**Figure 9:**
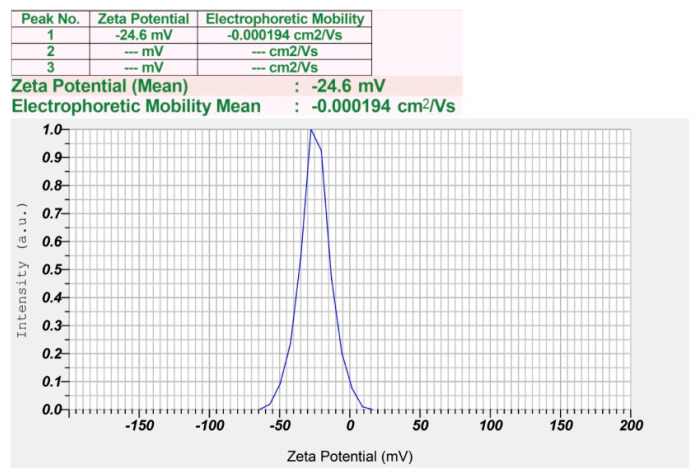
Zeta potential analysis of synthesized P-AgNPs from leaf extract of *P. alba*.

The crystalline nature and composition of AgNPs and the phase purity of synthesized P-AgNPs were analyzed by XRD. The XRD graph (Figure 8) shows specific Bragg’s reflections with 2θ values at 111, 200, 220, and 311, which correspond to 38.08°, 44.18°, 64.41°, and 77.35°, respectively, in the JCPDS database of silver. These lattice plane values confirm the face-centered cubic crystalline nature of the synthesized P-AgNPs. The average particle size was 26.43 nm as calculated by Scherr’s equation. The surface charge potential and stability of the nanoparticle in the aqueous suspension was analyzed by the zeta potential. The zeta potential of synthesized P-AgNPs showed a sharp peak at −24.6 mV (Figure 9) and confirmed good stability.

### 3.2. Antimicrobial Activity of Synthesized AgNPs

**Figure 10:**
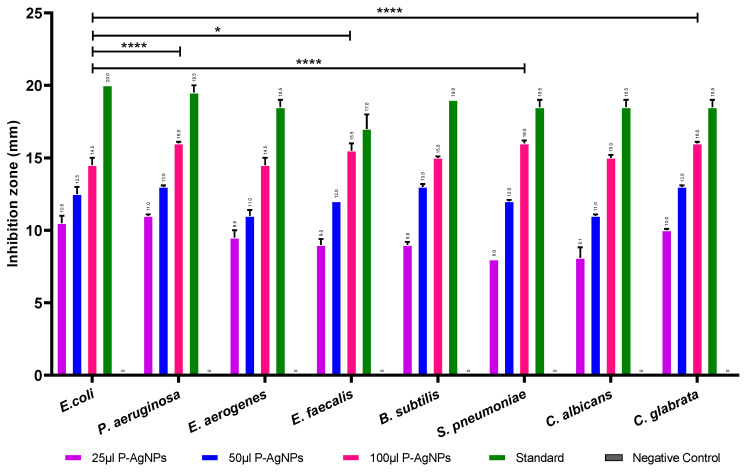
Antimicrobial activity of synthesized P-AgNPs against pathogenic microorganisms. Two-way ANOVA with multiple comparisons was performed to identify differences between groups. *p* < 0.05 (*), *p* < 0.001 (****).

Synthesized P-AgNPs with different concentrations of 25 µg, 50 µg, and 100 µg showed good antimicrobial activity against selected pathogenic microorganisms as depicted in Figure 10. The results are indicated as mm of inhibition zone diameters. Large inhibition zones ranging between 14.5 and 16 mm were noted for the higher concentration of 100 µg/mL P-AgNPs against all tested bacteria and lesser inhibition of bacterial growth with 25 µg/mL P-AgNPs. The antibacterial activity of 100 µg/mL P-AgNPs was compared among the bacteria. It was greatest against *S. pneumoniae* and *C. glabrata*, with an average inhibition zone diameter of 16 ± 0.2 mm (*p* = 0.0003, <0.0001) followed by *E. faecalis*, 15.5 ± 0.5 mm (*p* < 0.0001), *P. aeruginosa*, *B. subtilis* and *C. albicans* (15 ± 0.1 mm, *p* = 0.0453) whereas *E. coli* and *E. aerogenes* were least sensitive.

### 3.3. Anticancer Activity of Synthesized P-AgNPs

**Figure 11:**
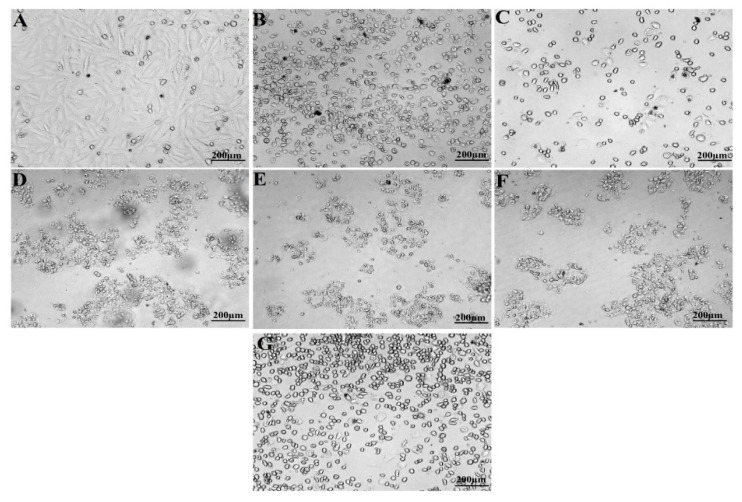
MTT assay of P-AgNPs from *P. alba* leaf extract against U118 MG cancer cell line. (**A**) Untreated cells, (**B**) Positive control, (**C**) 6.25 µg/mL, (**D**) 12.5 µg/mL, (**E**) 25 µg/mL, (**F**) 50 µg/mL, (**G**) 100 µg/mL.

**Figure 12:**
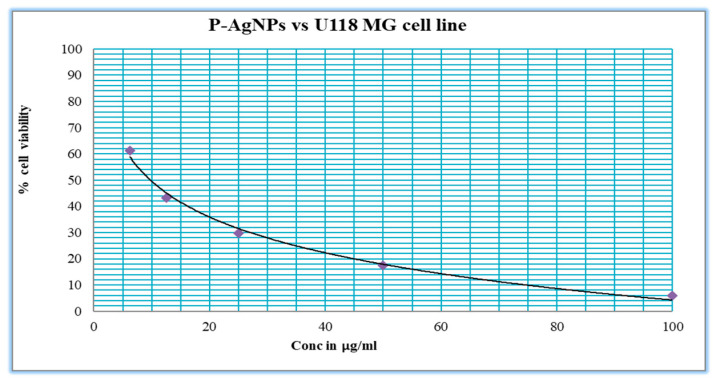
Graphical representation of percentage cell viability of *P. alba* AgNPs on U118 MG cancer cell line.

Synthesized P-AgNPs from *P. alba* leaf extract were treated against U118 MG cell lines (Human Brain Glioblastoma cell line, NCCS, Pune, India) for anticancer activity by MTT assay. It showed significantly anti-proliferative potential with IC_50_ concentration at 9.77 µg/mL after the incubation of 24 h, compared to the IC_50_ concentration of 6 µg/mL cisplatin that was used as a positive control (Figure 11 and Figure 12).

### 3.4. Apoptosis Assay of P-AgNPs by Flow Cytometry

**Figure 13:**
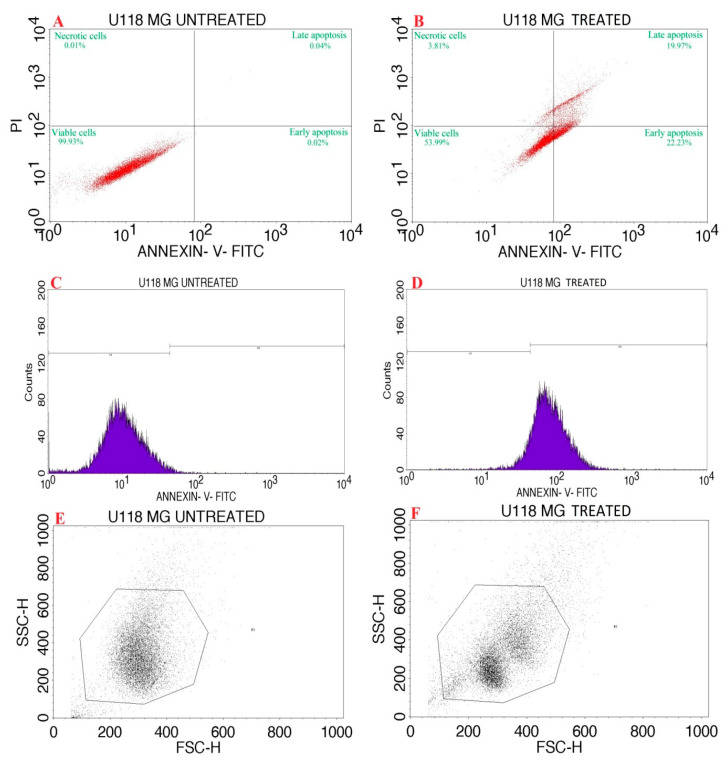
Flow cytometric analysis of *P. alba* AgNPs against U118 MG cancer cell line. Quadrangular plot of Annexin V/PI expression on U118 MG cells (**A**) Untreated cells, (**B**) AgNP-treated cells. Cell cycle of U118 cell line (**C**) Untreated, (**D**) AgNP-treated. Scatter plot of forward scatter (FSC) against side scatter (SSC) (**E**) Untreated cells, (**F**) AgNP-treated cells.

The synthesized P-AgNPs with an IC_50_ concentration 9.77 µg/mL was used for Annexin V/ PI expression study on U118 MG cancer cells. Figure 13A,B and Table 1 show the percentage of cells that underwent apoptosis, necrosis and the presence of viable cells. The P-AgNP-treated cells displayed apoptosis in 42.2% and necrosis in 3.81%, suggesting a decrease in cell viability. Figure 13C,D express that the treatment of P-AgNPs affects the cell cycle progression with cell cycle arrest in M1 (M1 represents negative expression, M2 shows positive expression over cancer cell line). Figure 13E,F represent the scatter plot of forward scatter (FSC) and side scatter (SSC) of AgNPs’ activity against GBM U118 MG cells.

## 4. Discussion

Nanotechnology is one of the most promising technologies of the 21st century [34]. AgNPs synthesis using plants offers a quick, environmentally friendly, practical, and cost-effective solution for pharmaceutical and biomedical applications [35]. The present study reported AgNPs formation using *Plumeria alba* leaf aqueous extract (P-AgNPs) with determination of its antimicrobial and anticancer activity. Green synthesis of nanoparticles was confirmed by a color change in the mixture of AgNO_3_ and plant extract. The color change of the reaction mixture was due to the presence of an NAD^+^ co-enzyme or ascorbic acid in the living cells, which were involved in the redox reaction and transforming ions from Ag^+^ to Ag^0^ in the reaction mixture [36]. The synthesis of nanoparticles is directly proportional to the availability of reducing and capping agents in the plant extract. The availability of the capping agents decreases as reaction time increases, resulting in proper stabilization and capping of nanoparticles [37]. A similar method of color change was reported by Sreelekha et al., where *Mussaenda frondosa* leaf synthesis of AgNPs showed a color change of mixture from pale yellow to reddish-brown [38].

UV-Vis spectroscopic analysis of synthesized mixture solution showed SPR absorption band at 403 nm because of the surface plasmon resonance excitation of nanoparticles. The SPR absorption band is due to the presence of free electrons in metal nanoparticles and their mutual oscillation in resonance with the wavelength of incident light. *P. alba* leaf extracts contains a wide range of phytochemicals, such as steroids, flavanoids, terpenoids, tannins, glycosides, saponins, sugars, etc., which favored the synthesis of P-AgNPs and can be considered as main constituents in the reduction, stabilization, and capping of nanoparticles [18]. Khan et al., reported a similar SPR UV sprectrum peak of synthesized AgNPs from aqueous leaf extract of *Trigonella foenum-graecum* at 410 nm [39]. Lopes et al. reported similar SPR peak of synthesized spherical AgNPs using the ALE of *Mimusops coriacea* at approximately 415 nm at room temperature with particles sized between 10 and 30 nm in diameter [40].

The peak areas in FTIR spectrum revealed the functional groups that might have involved in the reduction of Ag^+^ ions to AgNPs. FTIR spectra of *P. alba* AgNPs exhibited the presence of amines, alkanes, δ-lactones, alkenes phenols, alcohol groups, sulfoxide groups, and halo compounds; these compounds might have acted as stabilizing and capping agents for P-AgNPs synthesis. Although not investigated herein, the richness of phytochemicals in *P. alba* was identified previously, which provided nanosized particles in AgNPs fabrication [21]. The results were compared with Jyothi et al., whose FTIR spectrum of synthesized AgNPs from the leaf extracts of *Urtica dioica* Linn. showed the similar peaks and functional groups such as alcohol, phenol, aromatic compounds, alkanes, amines, and halo compounds [41]. Morphological study of green synthesized AgNPs using SEM analysis showed that the synthesized P-AgNPs were polydispersed, spherical in shape. Similar shapes were recorded by Anandalakshmi et al., where the SEM images of biosynthesized AgNPs from *Pedalium murex* leaf extract displayed even shape and spherical nature with particle size ranging from 20 to 50 nm [42]. Hemalata et al. also reported comparable shapes for the SEM images of biosynthesized AgNPs from a *Cucumis prophetarum* leaf extract [43]. The EDX analysis depicted a strong peak at 3 keV, which indicated the nanoparticles were composed of metallic silver particles. No other element was detected in EDX spectrum, which indicates the strength of the purity of P-AgNPs.

The TEM images of *P. alba* nanoparticles show their spherical appearance and poly-dispersal with a size range of 20.12 to 45.40 nm. Reportedly, synthesized AgNPs from aqueous extracts are finely dispersed; also, few of them have been recorded to be aggregated. This non-uniformity in the size and size distribution of nanoparticles is because of the heterogeneity of reducing agents in *P. alba* leaf aqueous extracts. The size and dispersity of nanoparticles are highly dependent on the reducing power of organic substances to reduce silver ions [37]. The results of synthesized P-AgNPs were compared with TEM images of nanoparticles synthesized from the leaf extracts of *Impatiens balsamina* and *Lantana camara*, which reported similar spherical shapes with a poly-dispersed nature where different molar concentrations of AgNO_3_ were used for the synthesis of nanoparticles [29]. The PDI value of synthesized P-AgNPs was determined as 0.25, which indicates the nanoparticles were poly-dispersed with variations in particle diameter. PDI is used to estimate the average uniformity of a particle solution, which ranges from 0 to 1. A larger size distribution of particles in the sample corresponds to larger PDI values. Aggregation of nanoparticles can also be indicated by PDI analysis along with the efficiency and consistency of particle surface modifications throughout the sample. A sample is considered mono-dispersed when the PDI value is less than 0.1 and poly-dispersed with multiple particle sizes when the PDI is more than 0.1 [44].

AFM analysis of reported P-AgNPs predominantly spherical in shape and poly-dispersed, and few of these seemed to be aggregated in nature. The size range of the nanoparticles was calculated to be 18.23 to 53.68 nm. The difference found in size ranges of TEM and AFM analysis might be due to difference in their imaging techniques [45]. The result obtained from AFM analysis of P-AgNPs can be compared to the results of SEM and TEM analysis, where the nanoparticles were observed to be spherically shaped, poly-dispersed, and seldom agglomerated. The results were compared with the synthesized Ds-AgNPs from the *Drosera spatulata* plant extract, which showed spherical- and crystalline-shaped particles with a size ranging from 5 nm to 30 nm and high negative zeta potential (−34.1 mV) [46]. It was reported that the aggregation of nanoparticles is mostly due to the capping of proteinaceous molecules around AgNPs. These proteinaceous-capping molecules form a multilayer of nano-silver protein by interacting with other capping molecules. Proteins are known to bind AgNPs covalently and form specific or nonspecific protein–protein interactions by attracting other capping molecules to induce agglomeration [37,47].

The crystalline nature of the synthesized P-AgNP was confirmed by X-ray diffractometry. The specific 2θ diffraction values clearly displayed peaks at 111, 200, 220, and 311, which corresponded to 38.08°, 44.18°, 64.41°, and 77.35°, confirming the face-centered cubic crystalline nature of *P. alba* AgNPs. The obtained peaks in XRD pattern clearly explained that the Ag^+^ ions had been completely reduced to Ag^0^ by the reducing and stabilization compounds in the aqueous extract under reaction conditions. There were some unassigned peaks in the chromatogram. The presence of sharp peaks in the chromatogram indicated proteins or some bioorganic compounds in the NPs during synthesis [42]. Similar results of crystalline, biosynthesized *Trapa natans* (*T. natans*) leaf extract AgNPs with a size range between 30 nm and 90 nm were reported by Saber et al., who obtain an XRD spectrum pattern that showed distinct diffraction peaks at 2θ angles of 38.19°, 44.37°, 64.77°, and 76.39°, indexed to the (111), (200), (220), and (311) Bragg’s reflection of the face-centered cubic structure of silver crystals, confirming their highly crystalline nature [48]. Similar results were reported by Krishna et al., whose synthesized AgNPs from a leaf extract of *Sansevieria roxburghiana* shows peaked values at 38°, 44°, 64°, 77°, confirming the crystalline face-centered cubic nature [49].

The zeta potential indicates the surface charge, stability and dispersion of nanoparticles present in the medium. The zeta potential of P-AgNPs displayed a sharp peak at −24.6 mV. Due to their negative value the biosynthesized nanoparticles were dispersed in colloidal solution, indicating their relative stability, whereas their incipient stability was ≤ −30 mV and ≥ +30 mV. Nanoparticles with zeta potentials less than −30 mV are considered anionic. The obtained zeta potential supported that P-AgNPs were likely to form aggregations with time due to lower repulsive force among nanoparticles and promote aggregation [37,46]. Sarkar and Kotteeshwaran and Rao et al. reported that nanoparticles synthesized from the leaf extract of *Punica granatum* and *Ocimum sanctum* showed a zeta potential value at −67.2 mV and −55.0 mV, respectively. The negative values suggest the stability of the nanoparticles [50,51].

The *P. alba* biosynthesized AgNPs demonstrated the concentration-dependent antimicrobial activity in the current study on the tested microbes represented higher microbial growth inhibition with 100 µg/mL P-AgNPs. It was found to exhibit the strongest antimicrobial potential against the Gram-positive bacteria (*S. pneumoniae* and *E. faecalis*) and the fungus *C. glabrata*, whereas it showed less activity against Gram-negative bacteria (*E. coli* and *E. aerogenes*). Similar findings were seen by a previous study conducted by Pallela et al., in which they synthesized AgNPs using an aqueous extract of the whole plant of *Sida cordifolia*, displaying characteristics of ultra-small and spherical-shaped (monodisperse) with a mean particle size of 3–6 nm, and assessed its antibacterial activity. The zone of inhibition formed by the synthesized *S. cordifolia* AgNPs (25 μg/mL) for Gram-positive bacteria *S. aureus* (19.50 ± 0.55) and *B. subtilis* (18.00 ± 0.63) recorded the highest values, followed by Gram-negative bacteria such as *K. pneumonia*, and *E. coli* [52] Another recent study demonstrated comparable antimicrobial activity of synthesized AgNPs from *Russelia equisetiformis* (R-AgNPs) and *Leucophyllum frutescens* (L-AgNPs) leaf extracts with a mean size of 151.7 nm for R-AgNPs and 112.9 nm for L-AgNPs and negative zeta potentials. Lower activity was noted against the Gram-negative microbe, *E. coli*, compared to Gram-positive one, *K. pneumonia* [53].

The charge and size of the synthesized nanoparticles play a critical role in antimicrobial activity, where the electrostatic force of attraction between the charge of the AgNPs and charge of the bacterial cell wall is necessary for effective antimicrobial activity, and with their small size, easy penetration via bacterial cell walls can be expected [54]. They induce cellular toxicity and oxidative stress that damage intracellular structures and modulation of signal transduction pathways, resulting in microbial death. AgNPs even have the capability to generate Ag^+^ ions from the nanoparticles, which are more toxic than AgNPs. It has been reported that the antimicrobial activity of nanoparticles is based on and proportional to the release of ions. Generally, the released ions are often accountable for toxicity [55]. When Ag+ ions are exposed to microbial cells, they are uniformly distributed, surrounding the cells with no specific localization. These generated positive ions penetrate the cells due to their electrostatic interaction with the cell wall, causing more toxicity to the cells [56]. Besides electrostatic attraction, the Ag^+^ ions interact with the sulfur containing proteins present in the cell wall and change the cell wall structure, leading to its disruption. The Ag^+^ ions can even alter the release and transport of K^+^ ions from the microbial cells. Besides affecting the transport activity, the increase in membrane permeability may have stronger effects, such as the loss of cellular contents by leakage, including nucleic acids, ions, sugars proteins, and occasionally cellular energy reservoirs called ATP [55,57]. The results were also compared with those of Mani et al., who synthesized AgNPs from the leaf extract of *Allium cepa*, for which the size of AgNPs was around 54.3 nm, and the zeta potential value was −19.1 mV. These were applied against pathogenic bacteria, displaying good activity against *E. coli*, moderate activity against *S. aureus* and *P. aeruginosa*, and less activity against *E. faecalis* [58] Sreenivasa et al., who also reported that poly-dispersed AgNPs, synthesized from *Paenibacillus sp*. NS-36 with a characteristic UV–visible (UV-Vis.) spectral peak at 397 nm and average size of 32.40 nm, showed a good zone of inhibition against *E. faecalis*, a moderate zone against *E. coli* and *S. aureus*, and the least zone of inhibition against *S. pneumonia* [59].

The biosynthesized P-AgNPs from the *P. alba* leaf aqueous extract expressed significant anticancer activity against the U118-MG cancer cell line with an IC_50_ of 9.77 µg/mL in our study. The results were compared with those of Mercurio et al., who used the compound fluvastatin against GBM cell lines U118-MG and U87-MG cell lines, which resulted in an IC_50_ value of 470 μM (193.45 µg/mL) and 880 μM (362.08 µg/mL), respectively, and an IC_50_ value of 90 μM (34.32 µg/mL) and 110 μM (41.95 µg/mL), respectively, for celecoxib [60]. Petricciuolo et al. reported 3-bromopyruvate applied against the U118 MG cell line resulted in an IC_50_ of 40 μM [61]. P-AgNPs were more active against the U118-MG cell line compared to fluvastatin, celecoxib, and 3-bromopyruvate, for which the obtained IC_50_ concentration of the synthesized P-AgNPs was 9.77 µg/mL against the U118-MG cell line. A similar result was obtained by a recent study, in which, compared to the control, an increased concentration of NP-Pt (0.14 µM/mL; 0.29 µM/mL; 0.65 µM/mL) resulted in linearly decreased cell vitality at a comparable level to cisplatin treatments in U87 GBM cells due the increased amount of cell death and induction of apoptosis that was detected in the Annexin V/PI assay [62]. Therefore, to investigate further inhibition mechanisms exerted, we carried out an Annexin V-FITC/PI apoptosis detection assay.

The confirmation of apoptosis induction was verified through an Annexin V-FITC–PI apoptosis detection assay, which is extensively used to discriminate living cells from both early and late apoptosis. It was noted that P-AgNPs resulted in a strong shift to early and late apoptotic cell populations, which indicates that P-AgNP extract exerts apoptosis cell death mode. The induction of the apoptotic signaling pathways to trigger cancer cell death is the main mechanism of most anticancer drugs. *P. alba* AgNPs showed 42% apoptosis and 3.81% necrosis and therefore decreased the cell viability. Similar results were also reported by Amini et al., who found that nanoparticles from an *Teucrium polium* extract showed significant enhancement of apoptosis on NALM-6 cells [63]. It could also be suggested that P-AgNPs enhance ROS generation, which, along with released Ag^+^, produces oxidative stress in the cell, causing damage to cell biomolecules, commencing cell structural changes, dysfunction, and DNA destruction, which could be demonstrated as apoptosis.

## 5. Conclusions

The present investigation reported a simple, eco-friendly green synthesis method for AgNPs using a plant extract of *P. alba*. The AgNPs’ synthesis was primarily noted by the change in color of the reaction mixture and confirmed with UV-Vis analysis. Biomolecules linked with the reduction, stabilization, and capping of AgNPs were recognized in the FTIR spectrum, and morphological details, such as their spherical shape and poly-dispersed nature were determined using SEM, TEM, and AFM analysis. The average size of nanoparticles was found to be 26.43 nm. The chemical composition of synthesized P-AgNPs was calculated by EDX analysis, and the XRD pattern showed an fcc crystal structure. Their biological activity was analyzed against six bacteria, two fungi, and the GBM U118 MG cancer cell line. The biosynthesized P-AgNPs displayed significant antimicrobial activity on these bacterial and fungal pathogens. Furthermore, this study successfully showed the potential cytotoxic nature of P-AgNPs towards the GBM U118 MG cancer cell line by inducing cell death by causing an increase in early and late apoptosis cells’ population. Therefore, this study provides an initial data that proposes P-AgNPs could be used as a potential source of new antimicrobial and anticancer compounds. These pronounced biological activities of P-AgNPs were influenced by the interactions and molecular structure of capping agents that stabilize them; therefore, it is a cost-effective and environmentally acceptable platform for producing therapeutic agents for the treatment of different human diseases. However, further research is required to identify NPs’ biological efficiency on large scale to identify and validate their cellular and molecular therapeutic mechanisms.

## Figures and Tables

**Table 1:** Table showing the % of cells of that underwent apoptosis and necrosis in untreated and test (PA) treated U118 MG cells in comparison to viable cells.

Quadrant	Necrosis	Late Apoptosis	Healthy Cells	Early Apoptosis
Label	UL	UR	LL	LR
Cell Control	0.01	0.04	99.93	0.02
PA	3.81	19.97	53.99	22.23

## Data Availability

The raw data used and/or analyzed during the current study will be available from the corresponding author on reasonable request.
